# Supplementary figures and images for: Derivation and internal validation of prediction models for pulmonary hypertension risk assessment in a cohort inhabiting Tibet, China
Source: eLife. 2024 Nov 11;13:RP98169. doi: 10.7554/eLife.98169 (PMC11554304; doi:10.7554/eLife.98169)

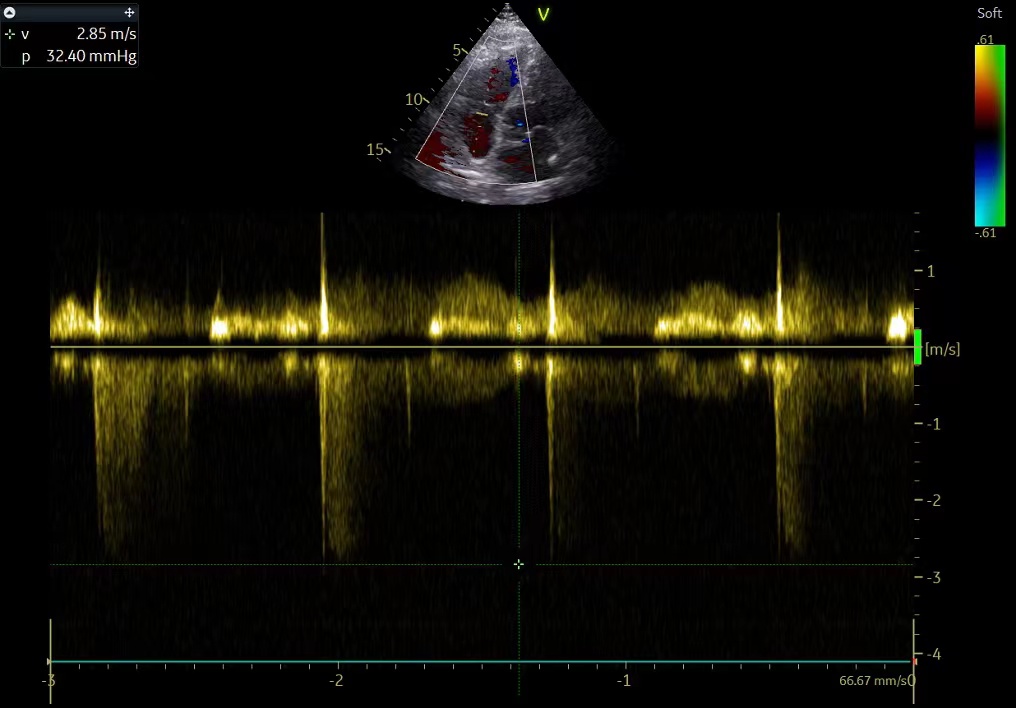

Supplement: Figure 2—source data 1. [file elife-98169-fig2-data1.zip › Figure 2/Figure 2. A.jpg]

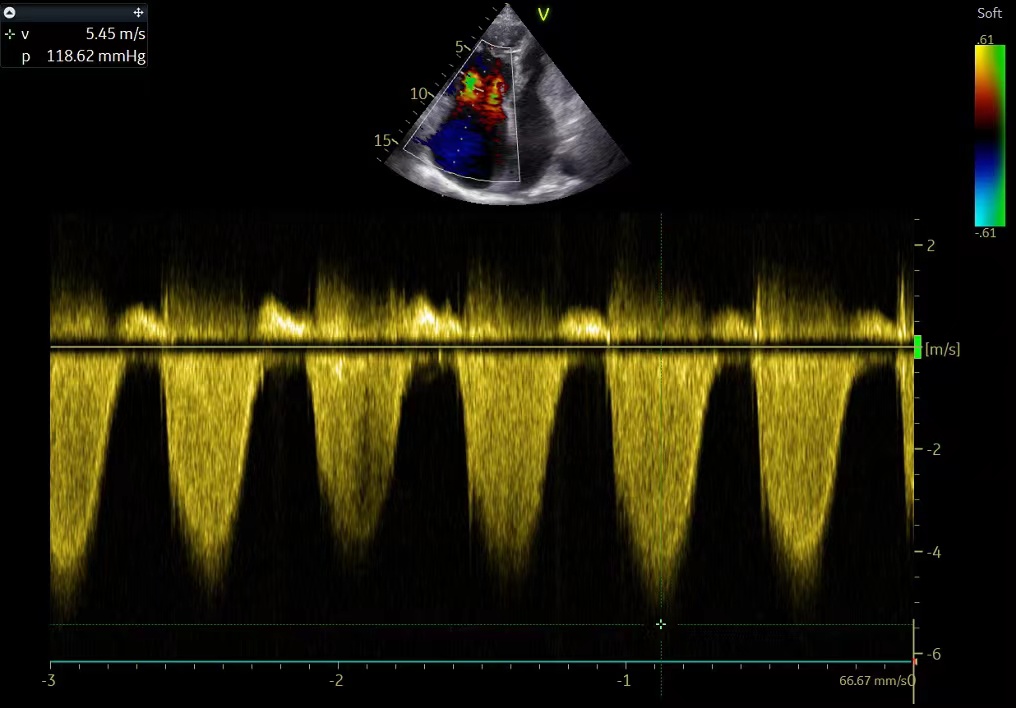

Supplement: Figure 2—source data 1. [file elife-98169-fig2-data1.zip › Figure 2/Figure 2.B.jpg]
